# The neonatal CNS is not conducive for encephalitogenic Th1 T cells and B cells during experimental autoimmune encephalomyelitis

**DOI:** 10.1186/1742-2094-10-67

**Published:** 2013-05-24

**Authors:** Petra D Cravens, Bernd C Kieseier, Rehana Hussain, Emily Herndon, Benjamine Arellano, Li-Hong Ben, Brenda C Timmons, Cyd Castro-Rojas, Hans-Peter Hartung, Bernhard Hemmer, Martin S Weber, Scott S Zamvil, Olaf Stüve

**Affiliations:** 1 Department of Neurology and Neurotherapeutics, University of Texas Southwestern Medical Center at Dallas, Dallas, TX, 75390-9036, USA; 2 Department of Neurology, Heinrich Heine University Düsseldorf, Düsseldorf, 40225, Germany; 3 Department of Pathology, University of Texas Southwestern Medical Center at Dallas, Dallas, TX, 75390, USA; 4 Hamon Center for Therapeutic Oncology, University of Texas Southwestern Medical Center at Dallas, Dallas, TX, 75390, USA; 5 Department of Neurology, Klinikum rechts der Isar, Technische Universität München, München, 81675, Germany; 6 Department of Neurology, University Medical Center, Georg August University, Göttingen, 37075, Germany; 7 Department of Neuropathology, University Medical Center, Georg August University, Göttingen, 37975, Germany; 8 Department of Neurology, University of California, San Francisco, CA, 94143, USA; 9 Program in Immunology, University of California, San Francisco, CA, 94143, USA; 10 Neurology Section, VA North Texas Health Care System, Medical Service, 4500 South Lancaster Rd, Dallas, TX, 75216, USA

**Keywords:** Age, Antigen presentation, Autoimmunity, Development, EAE, Experimental autoimmune encephalomyelitis, Human, Lymphocytes, Major histocompatibility complex, MHC, MS, Mouse, Multiple sclerosis, Rodent, T helper cells 17

## Abstract

Multiple sclerosis (MS) is thought to be a CD4^+^ T cell mediated autoimmune demyelinating disease of the central nervous system (CNS) that is rarely diagnosed during infancy. Cellular and molecular mechanisms that confer disease resistance in this age group are unknown. We tested the hypothesis that a differential composition of immune cells within the CNS modulates age-associated susceptibility to CNS autoimmune disease. C57BL/6 mice younger than eight weeks were resistant to experimental autoimmune encephalomyelitis (EAE) following active immunization with myelin oligodendrocyte glycoprotein (MOG) peptide (p) 35–55. Neonates also developed milder EAE after transfer of adult encephalitogenic T cells primed by adult or neonate antigen presenting cells (APC). There was a significant increase in CD45^+^ hematopoietic immune cells and CD45^+^ high side scatter granulocytes in the CNS of adults, but not in neonates. Within the CD45^+^ immune cell compartment of adults, the accumulation of CD4^+^ T cells, Gr-1^+^ and Gr-1^-^ monocytes and CD11c^+^ dendritic cells (DC) was identified. A significantly greater percentage of CD19^+^ B cells in the adult CNS expressed MHC II than neonate CNS B cells. Only in the adult CNS could IFNγ transcripts be detected 10 days post immunization for EAE. IFNγ is highly expressed by adult donor CD4^+^ T cells that are adoptively transferred but not by transferred neonate donor cells. In contrast, IL-17 transcripts could not be detected in adult or neonate CNS in this EAE model, and neither adult nor neonate donor CD4^+^ T cells expressed IL-17 at the time of adoptive transfer.

## Introduction

Multiple sclerosis (MS) is the most common inflammatory demyelinating disorder of the central nervous system (CNS) in humans with a presumed autoimmune pathogenesis [1]. A first-time diagnosis of MS is exceedingly rare during early childhood [2,3]. To date, the youngest patient reported with physician-diagnosed MS was 24 months old at the time of the initial clinical attack [4]. The youngest reported patient with acute demyelinating encephalomyelitis (ADEM), a self-sustained monophasic CNS autoimmune disease following an acute infection or immunization, was 15 months old [5]. Cellular or molecular mechanisms that confer relative disease resistance during infancy are unknown.

Activated myelin-reactive CD4^+^ Th1 cells are thought to have a central role in the pathogenesis of MS, and its prototypic animal model experimental autoimmune encephalomyelitis (EAE) [6]. CD4^+^ T cells are activated through recognition of linearized peptides presented in the context of major histocompatibility complex (MHC) II on antigen (Ag) presenting cells (APC) [7,8]. Ag presentation in the context of MHC II appears to be required at different stages of EAE and MS pathogenesis. Early studies suggested that the activation of T cells in secondary lymphoid organs facilitated their entry into the brain and spinal cord [9,10] but more recently, it was shown that CNS-specific T cells can enter the CNS without the requirement for antigen priming in peripheral lymphoid tissues [11]. While healthy CNS tissue is devoid of MHC II protein expression, circulating APC, including myeloid cells and B lymphocytes that have entered the perivascular spaces or parenchyma of the CNS from the peripheral blood constitutively express MHC II. After CNS injury, astrocytes [12,13] and microglial cells [14,15] can also upregulate MHC II in response to inflammatory mediators, including IFNγ, the signature cytokine of T helper 1 (Th1) cells [16]. MHC II-mediated Ag presentation results in reactivation of CD4^+^ T cells, and an amplified immune response [17]. Thus, the regulation and expression of MHC II genes are considered critical in MS and its animal model, EAE.

We utilized both the active immunization and adoptive transfer models of EAE to test the hypothesis that a differential composition of immune cells within the CNS modulates age-associated susceptibility to CNS autoimmune disease.

## Materials and methods

### Peptides

Mouse myelin oligodendrocyte glycoprotein peptide (MOG_p_) 35–55 [MEVGWYRSPFSRVVHLYRNGK] was synthesized by solid-phase Fmoc chemistry by QCB, Inc. (Hopkinton, MA, USA) and CS Bio (Menlo Park, CA, USA).

### Animals

C57BL/6 and B10.PL wild-type (wt) mice were purchased from the Jackson Laboratory (Bar Harbor, ME, USA). Vα2.3/Vβ8.2 TCR-transgenic mice provided by Dr Joan Goverman were bred in a specific pathogen-free facility at the University of Texas (UT) Southwestern. All protocols involving mice handling were approved by the UT Southwestern animal care facility.

### Induction of experimental autoimmune encephalomyelitis

To induce active EAE, C57BL/6 female mice were immunized subcutaneously with MOG_p35-55_ emulsified in an equal volume of complete Freund adjuvant (CFA) (DIFCO Laboratories, Detroit, MI, USA) in each flank. Mice were 4 days, 1, 2, 3, 4, 5, 6, 7, 8, and 20 weeks of age. Immediately after the immunization, and again 48 hours later, mice received an intravenous injection of pertussis toxin (Ptx) in PBS. All animals received an equivalent dose of Ag, adjuvants, and Ptx on a dose per weight basis: Per 20 g bodyweight, 100 μl of vaccine, containing 100 μg MOG_p35-55_ and 2 mg/ml mycobacterium, as well as 400 ng Ptx were administered.

For the induction of EAE by adoptive transfer, spleens from either 2-week-old or 8-week-old Vα2.3/Vβ8.2 TCR-transgenic mice [18], were removed and single-cell suspensions were prepared. Splenocytes were cultured in 24-well plates at 1 × 10^6^ cells/well with wt B10PL adult or neonatal irradiated splenocytes (3K Rads) at a ratio of 1:4. The cells were cultured in Roswell Park Memorial Institute (RPMI) medium 1640 with 10% FCS and stimulated with 6µg/ml myelin basic protein (MBP)_Ac1-11_ and 0.5ng/ml IL-12 in a 24-well plate for 72 hrs. Cells were washed with PBS and injected intraperitoneally (i.p.) into naive B10PL, adult (5 × 10^6^/200 µls) or neonatal (1 × 10^6^/50 µls) mice. Two independent experiments were conducted with a minimum of ten mice per group.

For all EAE experiments, individual animals were observed daily and clinical scores were determined as follows: 0 = no clinical disease, 1 = loss of tail tone, 2 = mild paraparesis, 3 = paraplegia, 4 = hindlimb and forelimb paralysis, 5 = moribund or death. Three independent experiments were conducted with a minimum of five mice per group.

### Proliferation assays

Splenocytes (5 × 10^5^ cells/well) from mice that had been immunized with MOG_p35-55_ 10 days prior to sacrifice, were cultured in the presence of MOG_p35-55_ in RPMI 1640, supplemented with 5 × 10^-5^ M 2-mercaptoethanol, 2 mM glutamine, 100 μg/ml penicillin, 100 μg/ml streptomycin, 10% FCS (HyClone, Logan, UT, USA). After 72 hrs of culture, cells were pulsed with 1 μCi (^3^H)-thymidine and harvested 16 hrs later.

For assessment of Ag presentation by APC of various ages, splenocytes were γ-irradiated (33 Gy), and plated at 5 × 10^5^ cells/well with 1 × 10^4^ MOG_p35-55_-specific CD4^+^ T cells, and increasing doses of MOG_p35-55_. For generation of MOG_p35-55_-specific T cells, spleens were removed 0 to 14 days after immunization, and splenocytes were cultured in the presence of MOG_p35-55_ (25 μg/ml) and IL-2 (25 IU q3d). Every 14 days, T cells were washed, counted, and re-stimulated with APC and Ag for a minimum of three times. The purity of CD4^+^ T cells was analyzed by flow cytometry prior to each experiment, and approached 95% (data not shown). Cultures were pulsed with (^3^H) thymidine at 48 hrs and harvested 16 hrs later. The mean cpm ± SD of (^3^H) thymidine incorporation was calculated for triplicate cultures.

### B cell adoptive transfer experiments

CD19^+^ B cells were negatively sorted by magnetic separation (Stem Cell Technologies, BC, Canada) with a purity of >85% from spleens of 8- to 12-week-old donor mice that had been actively immunized for EAE with 50 µg of mouse rMOG in CFA 10 days previously. Then, adjusting for body weight, 1 × 10^6^ B cells were transferred i.p. into 4-day-old neonates and 5 × 10^6^ B cells into 8-week-old adult recipients. At this time recipient mice were immunized with CFA/MOG_p35-55._ A week later, 3 × 10^6^ B cells from adult donors immunized as described above were transferred i.p. to both neonates and adult recipients. Mice were monitored daily for clinical signs of disease as described previously.

### *In vitro* T helper cell differentiation

Splenocytes were prepared from naïve 2- and 8-week-old mice and CD4^+^CD62L^+^ T cells were sorted on the FACSAria (purity was > 98%). *In vitro* polarization of T cells (0.25 × 10^6^ cells/well in 2ml complete RPMI) was done in 24-well plates coated with anti-CD3 (1 mg/ml) and anti-CD28 (10 mg/ml) (BD Biosciences, San Jose, CA, USA) as previously described [19]. For T cell polarization RPMI was supplemented as follows: 2 mg/ml anti-IFN-γ (R46A2) for Th0, 5 ng/ml IL-12 for Th1, 10 ng/ml IL-4, and 5 mg/ml anti-IFNγ for Th2 and 25 ng/ml IL-6, 0.5 ng/ml TGF-β, 10 ng/ml IL-1β and 10 ng/ml TNF-α for Th17. On day 3 cells were split into fresh antibody-coated plates and 1 ml of fresh RPMI supplemented with cytokines was added to the appropriate wells: 10 U/ml IL-2 and 2 μg/ml anti-IFNγ (R46A2) for Th0, 10 U/ml IL-2 and 5 ng/ml IL-12 for Th1, 10 ng/ml IL-4 for Th2 and 25 ng/ml IL-6, 0.5 ng/ml TGF-β, 10 ng/ml IL-1β and 10 ng/ml TNF-α for Th17. At 48 and 72 hrs of the second stimulation culture supernatants were collected and cytokine ELISA performed as described below. All monoclonal antibodies (mAb) and cytokines were purchased from R & D Systems (Minneapolis, MN, USA).

### Enzyme-linked immunosorbent assay

Cell culture supernatants from experiments described above were collected at 48- and 72-hr time points for cytokine analysis as previously described [9,10]. Quantitative ELISA for IL-17 and IFNγ was performed using paired mAb specific for corresponding cytokines as per manufacturer’s recommendations (BD Biosciences or R&D Systems). The results of ELISA assays are expressed as an average of triplicate wells ± SD. The SOFTmax ELISA plate reader and software was used for data analysis (Molecular Devices Corporation, Sunnyvale, CA, USA).

### Flow cytometry

Mice were perfused via the left ventricle with cold PBS and brains, spinal cords, and spleens were harvested. Tissues were pressed through a 70-μm nylon mesh cell strainer. Splenocytes were treated with RBC lysing buffer (Sigma-Aldrich, St. Louis, MO, USA). CNS cells from all mice in each experimental group were pooled and processed as previously described [11]. In brief, CNS cells were washed twice in 37% Percoll and CNS mononuclear cells were isolated by centrifugation at 2118 × *g* for 15 minutes at 22°C, over a 30/70% Percoll gradient. The interphase cells were collected, washed with 0.5% BSA/PBS, re-suspended in complete RPMI 1640, and counted. For flow cytometry, the following mAb were used: anti-CD3-Pacific Blue (500A2), anti-B220-PE (RA3-6B2), anti-CD11c-APC (HL3), anti-Gr1-APC-Cy7 (RB6-8C5), all from BD Biosciences; anti-CD11b PerCp-Cy5.5 (M1/70), anti I-A^b^ PE-Cy5 (M5/114-152), anti-CD45-PE-Cy7 (30-F11), anti-CD19-Alexa Fluor 700(1D3), biotinylated anti-pan NK (DX5), all from eBiosciences (San Diego, CA, USA); anti-CD4-PE-Texas Red (MCD0417) and anti-CD8-Pacific Orange (MCD0830), both from Invitrogen (Grand Island, NY, USA); and biotinylated anti-PDCA-1 from Miltenyi (Auburn, CA, USA). Biotinylated mAb were revealed with SA-Q Dot 655 from Invitrogen. Cells were re-suspended in staining buffer (4% FCS and 0.1% sodium azide in PBS) and Fc receptors blocked with anti-CD16/32 (BD Biosciences) for 15 minutes at 4°C before staining with mAb for 30 minutes at 4°C. Cells were washed, re-suspended in staining buffer, and fixed in 1% paraformaldehyde. Up to 500,000 events were acquired on a FACSAria (BD Biosciences) and analyzed using FlowJo software (Tree Star, Ashland, OR, USA).

### RNA isolation and quantitative real-time PCR (QRT-PCR)

Neonatal and adult animals were sacrificed on day 10 post immunization with CFA/MOG_p35-55_. Following 1.5% Avertin overdose (400 mg/kg for adults), animals were transcardially perfused with 20 ml ice cold PBS and the CNS was removed. Total RNA was extracted from CNS homogenates using standard techniques [20]. Taqman gene expression assays and the Step One Plus (Applied Biosystems, Foster City, CA, USA) were used to detect IFNγ, IL-10, IL-12 and IL-23. Relative gene expression between unimmunized mice (naïve) and mice immunized for EAE was determined using the ddCT method (Applied Biosystems User Bulletin #2). The dCt was normalized to the housekeeping gene ribosomal 18s rRNA, and the ddCt was normalized to the average of the naïve neonatal CNS dCts.

### Ki-67 staining

Ki-67 staining was performed to determine the number of proliferating cells in the CNS. Following fixation in 10% formalin, brains and spinal cords from naïve and immunized mice were serially sectioned. Tissues were embedded in paraffin, cut at 4 μm thickness, mounted on Fisher Brand Superfrost Plus glass slides, and stained with H&E (Fisher Scientific, Pittsburgh, PA, USA). Deparaffinization and staining with rabbit monoclonal Ventana anti-Ki-67 clone 30–9 was performed on the Ventana Benchmark XT automatic immunostainer with an ethylenediaminetetraacetic acid (EDTA)-based retrieval system (Ventana Medical Systems, Tucson, AZ, USA). The antibody was used at a concentration of 2 μg/ml, and immunoreactivity was detected with Ultraview horseradish peroxidase/diaminobenzidine. For analysis, the percentage of Ki-67-immunoreactive cells was calculated for selected mice using a Glasgow cell-counting graticule at 20× magnification with a Nikon Labophot-2 at a magnification of 20×. The thalamus location was chosen for analysis to avoid counting proliferating neuroglial precursors present in brains of immature mice. Ten fields were quantified for each mouse, and the mean was used for comparison.

### Confocal microscopy

Following fixation in 4% paraformaldehyde for at least 2 hrs, tissues were stored in 2% sucrose at 4°C until cutting. The brains were coronally sectioned and after embedding in Tissue-tek OCT Compound, the tissues were snap frozen in liquid nitrogen. Two 6 μm-thick sections were cut from each brain with a freezing microtome and mounted on Fisherbrand Superfrost Plus glass slides (Fisher Scientific, Pittsburgh, PA, USA). Tissue sections were then fixed in 10% formalin and rinsed in tap water. One set of tissues was stained with H&E and prepared for light microscopy, and the other set was prepared without staining for fluorescent microscopy. Areas of interest were captured on digitized images using a Leica TCS SP5 confocal microscope with the 63× objective and analyzed by ImageJ 1.35s software (public domain; http://rsbweb.nih.gov/ij/).

### Statistical analysis

All experiments were repeated at least twice. Correlations between continuous and categorical variables were assessed using the Mann-Whitney *U*-test. The means of two normally distributed samples were compared by Student *t*-test. All other statistical comparisons between groups were examined using one-way multiple range analysis of variance (ANOVA) for multiple comparisons or Student-Newman-Keuls multiple comparison test. A *P*-value <0.05 was considered significant. Data are given as mean ± standard error.

It is generally accepted that 10 mice per treatment group are required to test the effect of reagents in active immunization EAE. This number was determined through power analysis. Through testing the null hypothesis (that the mean disease severity will be equal among the two treatment groups), it is possible to attain >80% power with the following assumptions: the criterion for significance (alpha) has been set at 0.050; the test is two-tailed, which means that an effect in either direction will be interpreted; this computation assumes that the mean difference is 1.7 (corresponding to mean EAE scores of 1.7 versus 0.0; or 2 versus 3.7) and the common intra-group SD is 1.0. This minimum significant difference of 1.7 was selected from prior observations that repeatable effects were associated with changes in mean disease score of between 1.5 and 2.0. Thus, this work assumes that effects smaller than 1.7 would not be of clinical or substantive significance and that differences in mean clinical score of 1.7 can be anticipated. A second goal of this study was to estimate the mean difference between the two populations. On average, a study of this design would enable us to report the mean difference with a precision (95.0% confidence level) of plus/minus 1.13 points. For example, an observed difference of 1.7 between treatment groups would be reported with a 95.0% CI of 0.57 to 2.83. The precision estimated here is the median precision. Precision will vary as a function of the observed SD (as well as sample size), and in any single study will be narrower or wider than this estimate. All analyses were performed with Prism 5 for Windows (Graphpad, La Jolla, CA, USA).

## Results

### Susceptibility to experimental autoimmune encephalitis is age-associated

**Figure 1:**
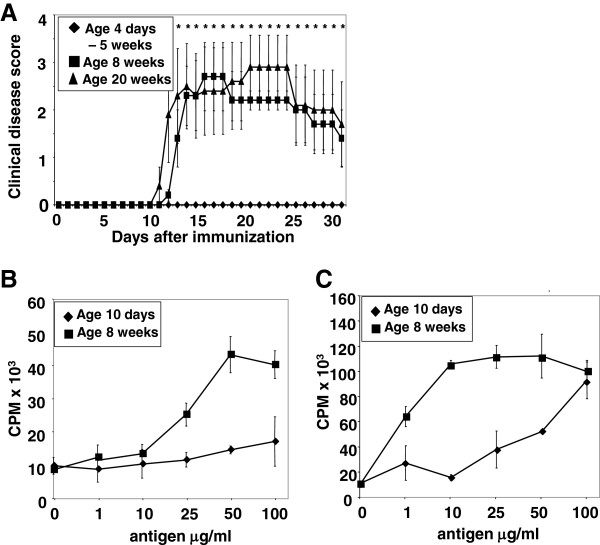
**Age-dependent disease susceptibility to experimental autoimmune encephalomyelitis.** (**A**) C57BL/6 mice (H-2^b^) between the ages of 4 days and 20 weeks were immunized with myelin oligodendrocyte glycoprotein (MOG)_p35-55_ to actively induce experimental autoimmune encephalomyelitis (EAE). Animals younger than 8 weeks were resistant to EAE. There was no difference in disease severity between 8- and 20-week-old mice, but both these groups are significantly different from mice younger than 8 weeks. (**B**) Age-associated capability of splenocytes to present antigen. The 10-day-old splenocytes fail to proliferate in response to antigen (Ag), whereas the proliferative responses in 8-week-old mice are robust. (**C**) Decreased proliferative responses are also observed when gamma-irradiated splenocytes from 10-day-old mice present MOG_p35-55_ at low and intermediate doses to MOG_p35-55_-specific T cells. This relative deficiency in Ag presentation by irradiated splenocytes from 10-day-old mice can be compensated with high doses of Ag.

To examine age-associated susceptibility to CNS autoimmune disease, EAE was induced in C57BL/6 mice between the ages of 4 days and 20 weeks by immunization with MOG_p35-55_. Animals younger than 8 weeks were resistant to EAE (Figure 1A). There was no difference with regard to disease severity between 8-week-old and 20-week-old mice (Figure 1A), but both these groups were significantly different from mice younger than 8 weeks.

### The age of APC contributes to recall proliferative responses *in vitro*

Since Ag-specific CD4^+^ T cell responses depend on interaction with MHC II molecules expressed on APC, T cell responses were initially assessed with primary proliferation assays. The 12-day-old splenocytes failed to proliferate in response to Ag, whereas the proliferative responses in splenocytes from 8-week-old mice were robust (Figure 1B). In order to determine whether absent proliferative responses of immature splenocytes were due to deficiencies in the APC or T cell compartment, irradiated, naive splenocytes of different ages were used to present MOG_p35-55_ to a MOG_p35-55_-specific T cell line that had been generated from mature mice. At 48 hrs, decreased proliferative responses were observed at low and intermediate Ag doses when presented by APC from 12-day-old mice (Figure 1C). This relative deficiency in Ag presentation by APC from 12-day-old mice could be compensated with high doses of Ag (Figure 1C).

#### Peripheral APC of neonatal mice are capable of generating encephalitogenic T cells

**Figure 2:**
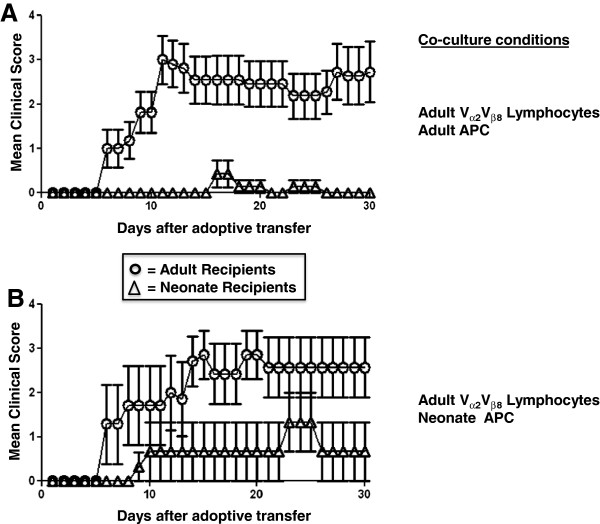
**Experimental autoimmune encephalomyelitis (EAE) disease course in adult mice is more severe than in neonates.** Adult and neonatal B10.PL recipient mice received 5 × 10 [6] (adults) or 1 × 10 [6] (neonates) adult Vα2Vβ8 transgenic T lymphocytes that had been re-stimulated *in vitro* for 72 hrs in the presence of MBP_Ac1-11_ peptide prior to adoptive transfer. Clinical scores shown are from at least two independent experiments. Error bars represent ± SD.

An adoptive transfer model employing Vα2.3Vβ8.2 TCR transgenic mice on the B10.PL background (H-2^u^) was utilized to examine the relative contribution of MHC II-restricted Ag presentation in the periphery and CNS on age-associated EAE susceptibility. In a first set of experiments, donor cells from adult Vα2.3Vβ8.2 TCR transgenic mice were primed *in vitro* with irradiated splenocytes from neonatal or adult B10.PL mice, and transferred into neonatal or adult recipients (Figure 2A and B). Adult recipients developed clinical disease earlier and more severely than neonatal recipients in both settings (Figure 2A and B). However, there was no difference in disease activity between neonatal or adult mice that received adoptively transferred adult CD4^+^ T cells primed by neonatal or adult APC *in vitro* (Figure 2A and B), indicating that neonatal APC in secondary lymphoid organs are generally capable of generating encephalitogenic T cells in the presence of high Ag concentrations. Similarly, adult recipients of adult CD4^+^ Vα2.3Vβ8.2 T cells co-cultured with either neonatal or adult APC developed clinical disease earlier and more severely than neonatal recipients (data not shown).

### The cellular composition of leukocytes within the CNS is age-associated

**Figure 3:**
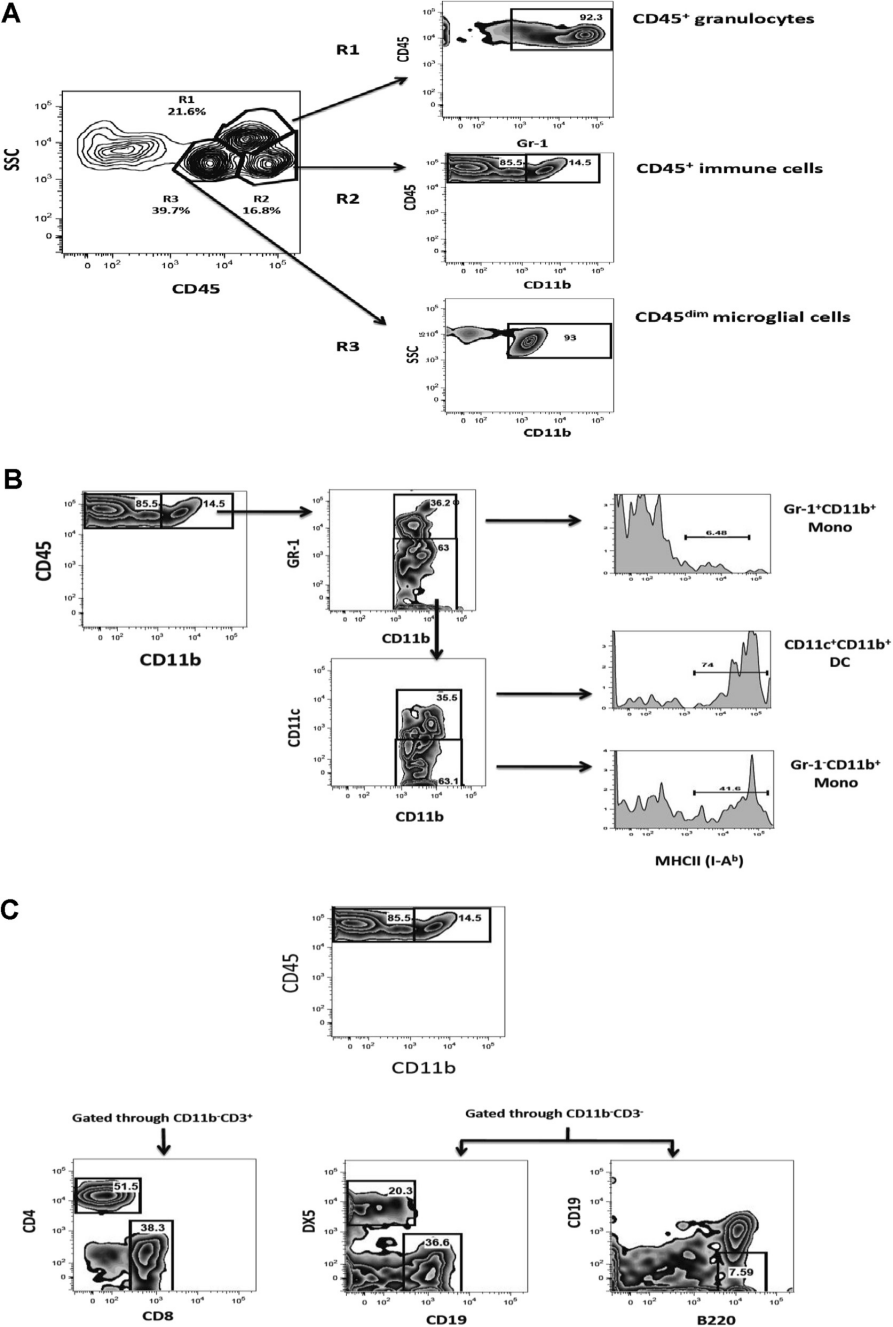
**Gating strategy to identify CD45+ hematopoietic immune cells that have accumulated in the central nervous system (CNS) of neonates and adult mice prior to onset of experimental autoimmune encephalomyelitis (EAE).** (**A**) At day 10 post immunization with complete Freund adjuvant (CFA)/ myelin oligodendrocyte glycoprotein (MOG)_35-55_, neonatal and adult mice were perfused and CNS cells isolated and prepared for flow cytometry. Cells were examined by side scatter (SSC) versus CD45 staining and three populations of CD45^+^ cells were identified; R1, CD45^+^ high SSC cells, which were Gr-1^+^ and represent granulocytes; R2, CD45^+^ hematopoetic immune cells, which were further divided into CD11b^+^ myeloid cells and CD11b^-^ non-myeloid cells containing lymphocyte subsets; and R3, CD45^dim^ microglia, which dimly express CD11b. Results are representative of three independent experiments. (**B**) Antigen-presenting cells (APC) within the CD45^+^CD11b^+^ cell population were identified by expression of Gr-1 to identify Gr-1^+^ monocytes, Gr-1^-^ monocytes, and CD11c^+^ DC. Histograms on the right represent staining for major histocompatibility complex (MHC) II using anti-I-A^b^. (**C**) T cells were identified in the CD45^+^CD11b^-^ fractions by expression of CD3, CD4 and CD8. CD19^+^ B cells and DX5^+^ NK cells were identified in the CD45^+^CD11b^-^CD3^-^ fraction. Plasmacytoid dendritic cells (PDC) were identified in the CD45^+^CD11b^-^CD3^-^ by expression of B220 or PDCA1.

**Figure 4:**
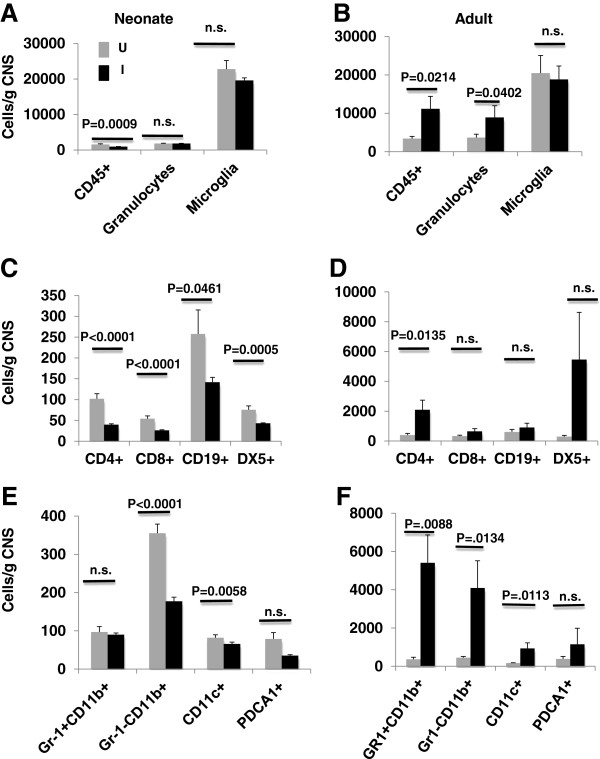
**Analysis of immune cell subsets in the central nervous system (CNS) post immunization with complete Freund adjuvant (CFA)/myelin oligodendrocyte glycoprotein (MOG)**_**p35-55**_**.** CNS mononuclear cells from unimmunized (gray bars) neonates (**A**, **C**, **E**) and adults (**B**, **D**, **F**) and from neonates and adults that had been immunized with CFA/MOG_p35-55_ 10 days earlier (black bars). Flow cytometry was used to examine the number of CD45^+^ immune cells, granulocytes and microglia/g of CNS (**A**, **B**), lymphocyte subsets (**C**, **D**), and myeloid subsets in panels (**E**, **F**). Figures are representative of data obtained from at least three independent experiments.

**Figure 5:**
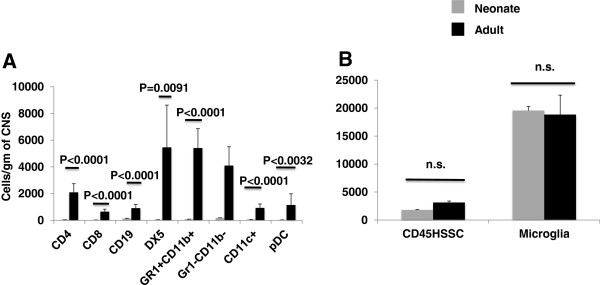
**Immune cells accumulate in the adult but not in the neonatal central nervous system (CNS) post-immunization with complete Freund adjuvant (CFA)/myelin oligodendrocyte glycoprotein (MOG)**_**p35-55**_**.** The number of CNS mononuclear cells/g of CNS from neonates (gray bars) and adults (black bars) are directly compared (**A**). There were significantly more CD45^+^ immune cell subsets in the adult versus the neonatal CNS by day 10 after immunization for experimental autoimmune encephalomyelitis (EAE).

To investigate the contribution of age to development of CNS autoimmunity, CNS from neonates (4 days old) and adult mice (6 to 8 weeks old) that had been immunized for EAE 10 days previously was examined for the presence of immune cells by multiparameter flow cytometry (Figure 3). Three populations of common leukocyte antigen CD45^+^ cells were identified in the CNS using side scatter (SSC) analysis (Figure 3A), Gr-1^+^ granulocytes (R1), CD11b^+^ myeloid and CD11b^-^ lymphoid immune cells (R2) and CD45^dim^ microglial cells (R3). A significant increase in CD45^+^ hematopoietic immune cells and CD45^+^ high SSC granulocytes was detected in the CNS of adults but not in neonates (Figure 4A and B). In fact, a significant decrease in the number of CD45^+^ hematopoietic cells occurred in the neonate CNS post immunization (*P* = 0.0009). This observed decrease was consistent within the CD45^+^ CNS lymphocyte population (gating strategy shown in Figure 3C) of neonates (Figure 4C), with a significant decrease in CD4^+^ and CD8^+^ T cells (p<0.0001), CD19^+^ B cells (p=0.0461) and DX5^+^ NK cells (*P* = 0.0005) after immunization for EAE. By contrast in the CNS of adult mice, CD4^+^ T cells significantly increased by day 10 post immunization (*P* = 0.0135) (Figure 4D). Within the myeloid cell compartment (gating strategy shown in Figure 3B) (Figure 4E and F), a significant decrease in Gr-1^-^CD11b^+^ monocytes (*P* <0.0001) and CD11c^+^ DC (*P* = 0.0058) was detected in neonatal CNS. The numbers of Gr-1^+^CD11b^+^ monocytes within the neonatal CNS were not impacted by immunization for EAE. In adults both Gr-1^-^ (*P* = 0.0088) and Gr-1^+^CD11b^+^ (*P* = 0.0134) monocytes and CD11c^+^ DCs (*P* = 0.0113) were significantly elevated post immunization. A direct comparison of cells/g of CNS in the immunized neonatal CNS versus immunized adult (Figure 5A) showed that CD4^+^ T cells as well as CD8^+^, CD19^+^, DX5^+^ NK, Gr-1^+^CD11b^+^ and Gr-1^-^CD11b^+^ monocytes, CD11c^+^ DC and pDC fail to accumulate in the neonatal CNS. The numbers of CD45+−HSSC granulocytes and CD45dim microglia in the neonatal and adult CNS were not statistically different at day 10 post immunization (Figure 5B).

### The increased number of Gr-1^+^CD11b^+^ monocytes in the neonatal CNS is not due to proliferation within the CNS

**Figure 6:**
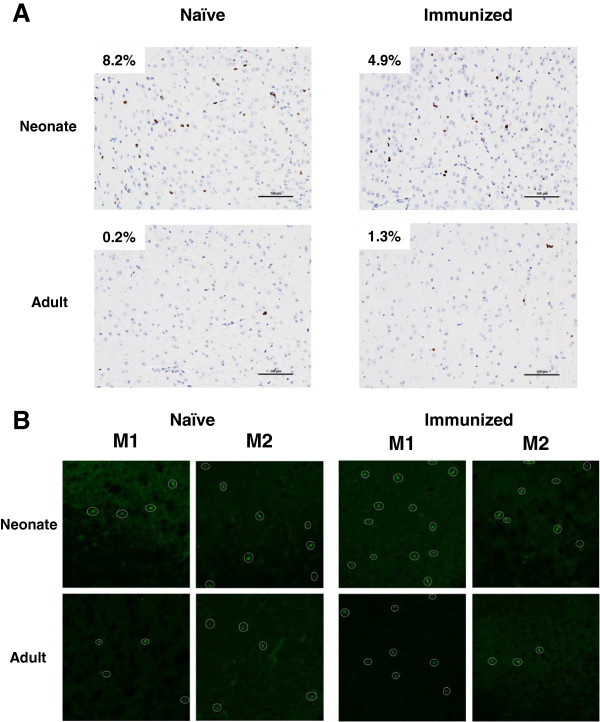
**Central nervous system (CNS) Ki-67 nuclear proliferation antigen counterstained with hematoxylin.** (**A**) Proliferating cells in the CNS of naive and immunized neonatal (top panels) and adult mice (bottom panels) are indicated by red circles (magnification ×20). (**B**) Analysis of naive and immunized neonatal and adult CNS by confocal microscopy. Circles indicate GFP^+^ cells. The results shown are examples of two individual mice (M1 and M2) per treatment group (magnification ×63). There was no significant change in the numbers of GFP^+^ cells between naive and immunized mice in either age group.

Since the numbers of all other immune cells examined in the neonatal CNS decreased at day 10 post immunization and only Gr-1^+^CD11b^+^ monocytes remained constant, we examined neonatal and adult CNS for expression of Ki-67 Ag to determine whether local expansion of immune cells contributed to the apparent accumulation within the adult CNS (Figure 6A). Consistent with our observations using flow cytometry, Ki-67 staining decreased two-fold (from 8.2% to 4.9%) in the neonatal CNS post immunization, whereas there was an increase in Ki-67 positive cells in the adult CNS (0.2% to 1.3%). These results indicate that proliferation within the CNS contributes to the accumulation of immune cells.

To examine more specifically the effect on myeloid cell numbers *in situ* in the CNS after immunization with CFA/MOG_p35-55_, neonatal and adult CX_3_CR1 ^GFP/+^mice in which cells of the myeloid lineage, including microglia, are GFP^+^[21], were evaluated by confocal microscopy for GFP-expressing myeloid cells in naïve and immunized animals (Figure 6B). The results of two individual mice (M1 and M2) per treatment group are shown. While the presence of Ki-67^+^ cells was decreased in the immunized neonates, the numbers of GFP^+^ cells observed in the CNS at day 10 post immunization remained stable in neonatal and adult mice.

### The differential cellular expression of MHC II is age-associated

**Figure 7:**
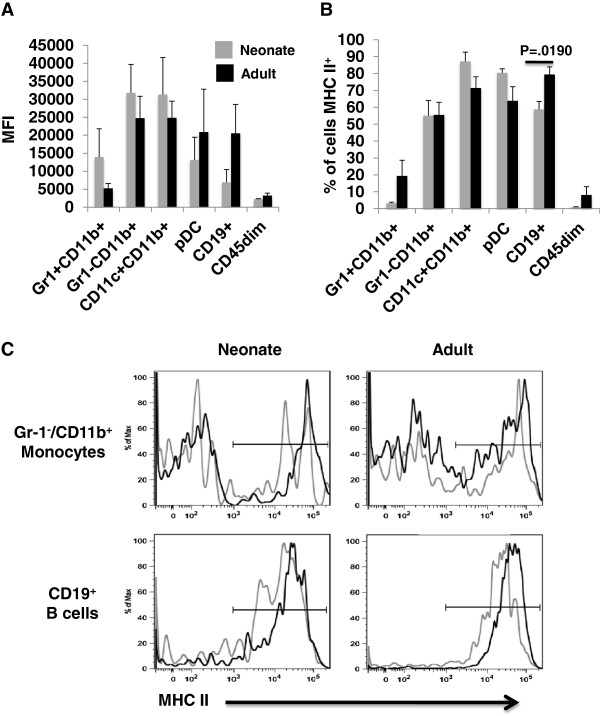
**Analysis of major histocompatibility complex (MHC) II expression on central nervous system (CNS) antigen-presenting cells (APC) subsets.** CNS mononuclear cells from neonatal (gray) and adult (black) mice immunized with complete Freund adjuvant (CFA)/myelin oligodendrocyte glycoprotein (MOG)_p35-55_ 10 days previously were isolated and prepared for flow cytometry. The median fluorescence intensity (MFI) of MHC II (**A**) and the percentage of each subset expressing MHC II (**B**) is shown. (**C**) Representative staining for MHC II on unimmunized (gray line) and immunized (black line) Gr-1^-^CD11b^+^ monocytes and CD19^+^ B cells is shown. An isotype-matched control mAb was used to identify positive staining events and set the marker. Figures are representative of data obtained from at least three independent experiments.

Since MHC II-mediated Ag-presentation results in re-activation of CD4^+^ T cells upon entry into the CNS, we examined CNS APC subsets from immunized neonatal and adult mice for expression of MHC II molecules. The differences in median fluorescence intensity (MFI) of MHC II staining were not statistically significant between neonates and adults for any of the APC subsets examined (Figure 7A). The percentage of CD19^+^ B cells that expressed MHC II in immunized adults was significantly different from neonate B cells (*p* = 0.019). No significant difference was observed in any of the other APC subsets examined. Representative staining for MHC II in unimmunized (gray) and immunized (black) on Gr-1-CD11b^+^ monocytes (top panels) and CD19^+^ B cells (bottom panels) is shown in panel C. Treatment of MS patients with B cell-depleting therapies has revealed a role for B cells in the pathogenesis of MS.

Since neonates immunized for EAE did not accumulate B cells in the CNS by day 10 post immunization and the percentage of neonate B cells expressing MHC II molecules was significantly reduced as compared to immunized adults, we examined whether EAE would develop in neonates if they received adoptively transferred B cells from immunized adults. Adult and neonatal C57BL/6 mice were immunized for EAE as described above. At days 3 and 10 post immunization CD19^+^ B cells were transferred i.p. and disease was followed for 25 days (data not shown). Adult animals developed EAE at day 12 post immunization, whereas neonates did not. Transfer of B cells isolated from immunized donors did not alter the onset of EAE nor disease severity suggesting that B cells are insufficient to induce or exacerbate early immune responses to MOG_p35-55_.

### The inflamed adult but not neonatal CNS promotes a Th1 environment

**Figure 8:**
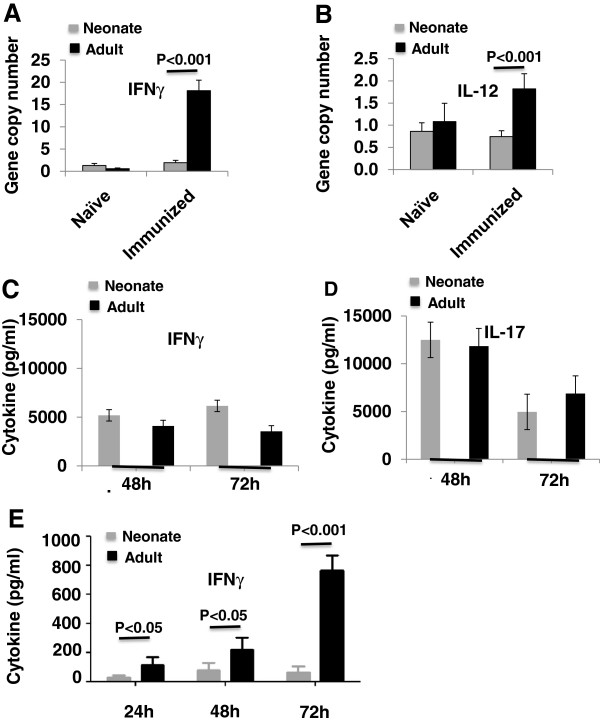
**IFNγ gene expression is modulated prior to experimental autoimmune encephalomyelitis (EAE) onset in the central nervous system (CNS) of immunized adults but not in neonates.** To examine whether the T cells from neonates have the potential to differentiate into T helper (Th) effector populations that contribute to CNS autoimmunity in adult mice, highly purified CD4^+^CD62L^+^ naive T cells from neonates and adults were differentiated *in vitro* in the presence of either Th1 or Th17 inducing conditions. Relative mRNA expression for (**A**) IFNγ, and (**B**) IL-12 is shown. Data represent an average ± standard error of the mean (SEM) for three to seven CNS tissues/group. Tissues were collected at day 10 post immunization for EAE. *In vitro*, neonatal CD4^+^ T cells are as capable as adult CD4^+^ T cells of producing (**C**) IFNγ under Th1 polarizing conditions, and (**D**) IL-17 under Th17 polarizing conditions. Supernatants were collected at 48 hrs and 72 hrs and the levels IFNγ and IL-17 were determined by ELISA. (**E**) To quantify IFNγ expression in neonatal and mature encephalitogenic CD4^+^ T cells, lymph node cells were prepared for adoptive transfer as described above [22]. IFNγ expression was determined by ELISA after 24, 48, and 72 hrs in culture. At all time points, adult CD4^+^ T cells expressed significantly more IFNγ than neonatal CD4^+^ T cells.

To determine whether there is a difference between neonates and adult mice in the cytokines present in the CNS at day 10 post immunization and before disease onset, total RNA was isolated from the CNS and real time PCR performed (Figure 8A, B). Immunized adults but not immunized neonates expressed IFNγ at day 10 post immunization with CFA/MOG_p35-55_ (Figure 8A). The relative gene expression for IL-12 (Figure 8B) was also significantly higher in the CNS of immunized adult mice than neonates. IL-10 and IL-17 transcripts were not detected in neonates or adults (data not shown). Therefore the neonatal CNS remains un-inflamed after immunization through a mechanism that does not involve IL-10.

One of the major triggers of inducible MHC II expression is IFNγ, which is expressed by CD4^+^ T cells, CD8^+^ T cells, natural killer (NK) cells, and natural killer T (NKT) cells. The source of IFNγ in EAE is likely activated CD4^+^ T cells, as the disease is induced through antigen presentation of MHC II-restricted Ag. To examine whether the T cells from neonates have the potential to differentiate into Th effector populations that contribute to CNS autoimmunity in adult mice, highly purified CD4^+^CD62L^+^ naive T cells from neonates and adults were differentiated *in vitro* in the presence of either Th1- or Th17-inducing conditions. Supernatants were collected at 48 hrs and 72 hrs and the levels IFNγ and IL-17 were determined by ELISA. *In vitro*, neonatal CD4^+^ T cells were as capable as adult CD4^+^ T cells of producing IFNγ under Th1 polarizing conditions (Figure 8C), and IL-17 under Th17 polarizing conditions (Figure 8D).

To quantify IFNγ expression in neonatal and mature encephalitogenic CD4^+^ T cells, lymph node cells were prepared for adoptive transfer as described above [22], and IFNγ expression was determined by ELISA assay after 24, 48, and 72 hrs in culture. At all time points, adult CD4^+^ T cells expressed significantly more IFNγ than neonatal CD4^+^ T cells (Figure 8E).

## Discussion

Several age-associated mechanisms have been proposed to explain the low disease incidence of MS in infants. Previous studies in EAE suggested that the relative lack of CNS myelination in the immature brain and spinal cord may contribute to the relative EAE resistance in immature rodents [23]. As immune responses against myelin autoantigens, including myelin basic protein (MBP) [24] and MOG [25], may be critical in MS pathogenesis, the formation of the myelin sheath around CNS axons may be an absolute requirement for CNS autoimmunity to occur. Even though CNS myelination in mice is completed at age 3 weeks, incomplete CNS myelination would not explain the results of our study, as neonates develop EAE after the adoptive transfer of encephalitogenic T lymphocytes.

It also has to be considered that the ascertainment of MS incidence and prevalence in very young infants is substantially more complex than in adolescents and adults. Among the most common clinical signs and symptoms in adult MS patients are fatigue, imbalance, pain, cognitive impairment, sexual and bladder dysfunction, and weakness [26]. It appears difficult, if not impossible, to detect some of these symptoms in infants under the age of two years.

In this study we show that neonatal mice are resistant to EAE and that this observed decrease in susceptibility is not associated with an absolute inability of neonatal T cells to develop into the Th1 and Th17 effector under optimized *in vitro* conditions. In light of our observations that neonatal peripheral APC acted as weaker APC both for proliferation of a differentiated T cell line, as well as for differentiation of naive T cells into IFNγ producing Th1 cells, it appears important to note that these data do not exclude that differences in peripheral APC compartments may contribute to EAE resistance of younger mice. Regardless, our findings highlight that resistance to EAE is primarily associated with a relative failure to accumulate immune cells within the CNS after immunization with CNS Ag, and immediately prior to disease onset. Specifically, we found that in contrast to the adult CNS where the numbers of CD4^+^ lymphocytes, granulocytes and myeloid APCs, such as Gr-1^+^ and Gr-1^-^ monocytes, and CD11c^+^ DC are increased by day 10 after immunization, in the neonatal CNS all lymphocyte and most myeloid subsets examined were significantly decreased. The numbers of granulocytes and Gr-1^+^ monocytes were unaffected after immunization.

Interestingly, no significant difference with regard to MHC II expression was observed between neonatal and adult mice in any CNS APC population other than CD19^+^ B cells. The number of CD19^+^ B cells that expressed MHC II in immunized adults was significantly higher compared to that in neonate B cells. This is an intriguing observation, as there is accumulating evidence that indicates that myelin-specific B cells and myelin-specific antibodies may have important roles in the pathogenesis of MS. A humoral component in MS has been implicitly recognized for decades [27-29], evidenced by inclusion of cerebrospinal fluid (CSF) oligoclonal bands and increased intrathecal immunoglobulin (Ig) G synthesis in MS diagnostic criteria [30,31]. Antibody deposition and immune complement activation associated with vesicular disintegration of the myelin membrane is present in most MS lesions [25,32,33], and autoantibody responses against many Ags can also be detected in the CSF of many MS patients [34]. B lymphocytes, of course, are much more than precursors of plasma cells and are very capable of presenting Ag to T cells [35]. The unique aspect of B cells as APCs derives from the expression of the high-affinity B cell receptor, which recognizes soluble, intact Ags. In addition, secretion of Fc receptor binding antibodies by activated B cells promotes opsonization and leads to enhanced APC function by DC. Adoptive transfer of B cells from adult donor mice immunized with rMOG did not increase disease susceptibility in neonatal recipients. Given that the autoantigen in this experiment was a linear peptide, it is quite possible that the majority of the transferred B cells were not antigen-specific.

We demonstrate that in the animal model of MS, EAE cannot be induced with a standard induction protocol in otherwise susceptible mice that are below a certain age. The results of our study are supported by published work by Massa *et al*., who showed that the degrees of EAE susceptibility in different strains of rats correlates with the quantity of MHC II expression within the CNS [36]. Specifically, it was demonstrated that fully susceptible Lewis rats (RT-1^b^) express much higher levels of MHC II in the CNS than fully resistant Brown Norway rats (RT-1^n^) [36]. This observation could be reproduced in EAE-susceptible SJL mice (H-2^s^) and EAE-resistant Balb/c mice (H-2^d^) [36], which indicates that this phenomenon may be of critical importance in determining susceptibility to CNS autoimmune disease in other mammalian species. Neither one of the prior two publications determined the cellular sources of MHC II expression within the CNS. Our group had previously demonstrated that Ag processing and presentation within the CNS is an absolute requirement for susceptibility to CNS autoimmune disease [37].

As mentioned above, B lymphocytes constitutively express MHC II. The MHC II transactivator (CIITA) is considered the master regulator of MHC II expression [38]. Although CIITA promoter III is viewed as the hematopoietic-specific promoter of CIITA, recent findings demonstrate that promoter IV is active in B lymphocytes and potentially contributes to the expression of CIITA and MHC II in these cells [39]. Thus, expression of IFNγ by T cells likely results in significant upregulation of MHC II by B lymphocytes. At day 10 after immunization, real time PCR revealed that the relative expression of IL-12 and IL-23 in both the neonatal and adult CNS was low. Transcripts for IL-10 were absent in the neonatal and adult CNS (data not shown). By contrast, the relative expression of IFNγ transcripts significantly increased in adults, but not in neonates, by day 10 after active immunization with CFA/MOG_p35-55_. The presence of IFNγ transcripts in the CNS immediately prior to disease onset may suggest that (1) peripherally activated CD4^+^ T cells are the main provider of this cytokine, and (2) IFNγ plays a critical role in the activation of local APCs and in the induction of MHC II on these cells, including on B cells. Overall, the Th1 environment is likely permissive for the reactivation of encephalitogenic Th1 lymphocytes that continue to migrate into the CNS from the peripheral blood.

We wanted to test the hypothesis that a differential composition of immune cells within the CNS modulates age-associated susceptibility to CNS autoimmune disease. The interpretation of investigation has some limitations. In essence, there are at least two compartments that determine susceptibility to CNS autoimmunity: (1) secondary lymphoid organs, where T cell activation in the context of an MHC II-restricted Ag presentation, as well as bystander activation of other immune systems occurs, and (2) the CNS itself, where re-activation of T cells and chemoattraction of other immune cells takes place. Many of our experiments were conducted in active EAE. In this model, immunophenotyping allows us to determine the composition of immune cells in different compartments during different ages. One disadvantage of this model is that specific pathogenic mechanisms that lead to a certain cellular composition can only be speculated upon. The adoptive transfer EAE model dissects the role of antigen-specific T cells and APC of different age groups in the periphery in susceptibility to CNS autoimmune disease. In this model, the requirement for T cell re-activation within the CNS is reduced. The disadvantage of the adoptive transfer EAE model is the focus on antigen-specific CD4^+^ T cells, whereas the role of other immune cells in the periphery and the CNS is less clear. Thus, with regard to the human disease MS, the adoptive transfer EAE model appears even more artificial than active EAE. In addition, there are experimental and ethical challenges in conducting true neonatal adoptive transfers into adult recipients, as T cells at age 16 days already assume characteristics of adult cells.

In summary, our data suggest that the neonatal CNS is less conducive to autoimmunity than the adult CNS. As the EAE model that was utilized in our experiments uses an MHC II-restricted myelin autoantigen to induce disease, the differential numbers of MHC II-positive B cells in the brains of neonatal and adult mice is intriguing. While our data are not entirely conclusive in this regard, they provide a rationale for a mechanism to explain the decreased risk of developing CNS autoimmune disease at an early age.

## Abbreviations

ADEM: Acute demyelinating encephalomyelitis; Ag: Antigen; APC: Antigen presenting cells; BSA: Bovine serum albumin; CFA: Complete Freund adjuvant; CIITA: MHC II transactivator; CNS: Central nervous system; CSF: Cerebrospinal fluid; DC: Dendritic cells; EAE: Experimental autoimmune encephalomyelitis; ELISA: Enzyme-linked immunosorbent assay; FCS: Fetal calf serum; GFP: Green fluorescent protein; H&E: Hemotoxylin and eosin; IFN: Interferon; IL: Interleukin; i.p.: Intraperitoneally; Ig: Immunoglobulin; mAb: Monoclonal antibodies; MBP: Myelin basic protein; MFI: Median fluorescence intensity; MHC: Major histocompatibility complex; MOG: Myelin oligodendrocyte glycoprotein; MS: Multiple sclerosis; NK: Natural killer; PBS: Phosphate-buffered saline; Ptx: Pertussis toxin; QRT-PCR: Quantitative real-time polymerase chain reaction; RPMI: Roswell Memorial Park Institute; SSC: Side scatter; Th1: T helper 1; TNF: Tumor necrosis factor; UCSF: University of California San Francisco; UT: University of Texas; Wt: Wild-type

## Competing interests

The authors declare that they have no competing interests.

## Authors’ contributions

PDC: Designed research, performed research, analyzed data, wrote the manuscript. BCK: Designed research, analyzed data, wrote the manuscript. RH: Designed research, performed research, analyzed data. EH: Designed research, performed research, analyzed data, wrote the manuscript. BA: Analyzed data, wrote the manuscript. LHB: Analyzed data, wrote the manuscript. BCT: Performed research, analyzed data. CCR: Performed research, analyzed data. HPH: Designed research, performed research, wrote the manuscript. BH: Designed research, wrote the manuscript. MSW: Designed research, performed research, analyzed data, wrote the manuscript. SSZ: Designed research, performed research, analyzed data. OS: Designed research, performed research, analyzed data, wrote the manuscript. All authors read and approved the final manuscript.
